# Novel Dual-Constraint-Based Semi-Supervised Deep Clustering Approach

**DOI:** 10.3390/s25082622

**Published:** 2025-04-21

**Authors:** Mona Suliman AlZuhair, Mohamed Maher Ben Ismail, Ouiem Bchir

**Affiliations:** Department of Computer Science, College of Computer and Information Sciences, King Saud University, Riyadh 11543, Saudi Arabia; mbenismail@ksu.edu.sa (M.M.B.I.); obchir@ksu.edu.sa (O.B.)

**Keywords:** semi-supervised clustering, fuzzy clustering, soft constraints, dual constraints, deep clustering

## Abstract

Semi-supervised clustering can be viewed as a clustering paradigm that exploits both labeled and unlabeled data to steer learning accurate data clusters and avoid local minimum solutions. Nonetheless, the attempts to refine existing semi-supervised clustering methods are relatively limited when compared to the advancements witnessed in the current benchmark methods in fully unsupervised clustering. This research introduces a novel semi-supervised method for deep clustering that leverages deep neural networks and fuzzy memberships to better capture the data partitions. In particular, the proposed Dual-Constraint-based Semi-Supervised Deep Clustering (DC-SSDEC) method utilizes two sets of pairwise soft constraints; “should-link” and “shouldNot-link”, to guide the clustering process. The intended clustering task is expressed as an optimization of a newly designed objective function. Additionally, DC-SSDEC performance was evaluated through comprehensive experiments using three real-world and benchmark datasets. Moreover, a comparison with related state-of-the-art clustering techniques was conducted to showcase the DC-SSDEC outperformance. In particular, DC-SSDEC significance consists of the proposed dual-constraint formulation and its integration into a novel objective function. This contribution yielded an improvement in the resulting clustering performance compared to relevant state-of-the-art approaches. In addition, the assessment of the proposed model using real-world datasets represents another contribution of this research. In fact, increases of 3.25%, 1.44%, and 1.82% in the clustering accuracy were gained by DC-SSDEC over the best performing single-constraint-based approach, using MNIST, STL-10, and USPS datasets, respectively.

## 1. Introduction

Typically, supervised learning approaches require accurate labeled collections of data to build accurate inference models [1]. However, the increasing size of data repositories has rendered the labeling process even more time-consuming and labor-intensive. On the other hand, contending with only relatively small datasets makes the intended models prone to overfitting. This promoted researchers’ efforts to investigate further unsupervised machine learning approaches. The motive is to cope better with unlabeled datasets as they relax the need for labeled training sets. In fact, cluster analysis has been employed to address problems in various areas, such as data mining, pattern recognition, and computer vision [2,3]. The goal of clustering consists of discovering homogeneous groups of data objects. Specifically, the instances assigned to the same cluster should exhibit similar features in comparison to the instances that belong to other clusters. Thus, clustering similar data samples together reveals the true data partition and provides insights into the hidden patterns beneath the distinct categories. Conventional clustering techniques are typically preceded with feature extraction to encode and capture data properties that reveal hidden patterns. However, this feature extraction can result in a high-dimensional representation of data, which can lead to a curse of dimensionality problem [4]. Consequently, several dimensionality reduction techniques [5,6] have been introduced to transform the data into a lower dimensionality feature space. Despite these advances, clustering data with complex latent structures remains challenging using existing methods [2].

The rise and rapid advancement of deep neural networks (DNNs) have triggered a reinvestigation of the research related to clustering. In particular, researchers have exploited the deep networks’ capability to automatically extract the most related data features and discard the need for feature engineering [2,7], for a more accurate clustering performance. Moreover, deep clustering paradigms aim to overcome challenges like handling nonlinear datasets, dealing with high-dimensional data, and reducing sensitivity to data noise [8,9].

Basically, early deep clustering works [10,11] considered feature transformation and clustering as two separate processes. Specifically, the data were first transformed into a new feature space and then fed into a clustering algorithm. Recently, deep clustering has improved to perform feature learning, transformation, and clustering of highly complex data in a joint manner [2]. Deep clustering approaches rely on deep neural network architectures [12], the network loss, and the clustering loss optimization [3]. This progress boosted the researchers’ efforts to inject some supervision to guide the clustering process, i.e., the semi-supervised learning paradigm. Specifically, semi-supervised learning uses both labeled and unlabeled data to train a model. In particular, it exploits prior knowledge and formulates it as constraints to ease the learning process. Pairwise constraints are a type of instance-level constraints that are used to represent prior knowledge. In fact, such constraints are induced by the perceived similarities between data instances. Several semi-supervised deep clustering approaches were recently proposed, including works based on graph clustering [13,14], density-based clustering [15], multi-view clustering [16], KL divergence clustering [17,18], and contrastive clustering [19]. While the side information in [20] is included in the model as a prior on the class proportions, the must-link and cannot-link pairwise constraint pairs in [21] are employed to define the thresholds for distinguishing between similar and dissimilar data points.

Despite ongoing efforts by researchers, the body of work on state-of-the-art semi-supervised deep clustering approaches remains considerably limited in comparison to the research conducted on unsupervised approaches [22]. In this paper, we propose DC-SSDEC to overcome the limitations of the current approaches. DC-SSDEC relies on a deep neural network architecture for feature learning and performs fuzzy membership-based clustering to mine the true partition of the data. In particular, DC-SSDEC introduces two sets of soft constraints within a novel objective function. This function is devised to learn the concealed data clusters and optimize the deep neural network in a simultaneous manner.

Additionally, fuzzy logic has been used in clustering to better represent data groupings [17,23]. This is attributed to the fact that fuzzy clustering allows individual data samples to belong to multiple clusters with varying probabilities, offering more flexibility. The fuzzy membership function assigns values between 0 and 1, indicating the similarity degree between a data sample and a cluster’s centroid. A higher probability suggests stronger confidence in the cluster assignment. Moreover, unlike overlapping clustering methods, the sum of membership values for a data instance must equal 1.

Furthermore, the majority of research in the literature on semi-supervised deep clustering utilizes “must-link”, and “cannot-link” pairwise constraints [24,25,26,27]. These constraints are considered hard as they enforce the data sample belonging to a certain cluster. On the other hand, soft “should-link” and “shouldNot-link” pairwise constraints are incorporated within the objective function of the proposed (DC-SSDEC) to express the supervision in hand. These constraints are formulated in a relaxing way in which the compliance with a constraint is not strictly obligated. The constraints are formulated in a relaxed manner, wherein strict compliance is not required. This flexible formulation enhances the applicability of the proposed approach to real-world data clustering tasks, where side information can be incomplete or unreliable, and thus cannot be firmly enforced.

## 2. Related Works

Different deep clustering frameworks based on various DNN architectures have been recently released [2]. Namely, deep clustering applications have adopted autoencoders (AEs), deep belief networks (DBNs) [28], convolutional neural networks (CNNs) [29,30], and generative adversarial networks (GANs) [31] as DNN architectures for feature learning and extraction. In fact, autoencoders have been widely used to tackle challenges relevant to deep clustering models [12,32,33,34]. Generally, AE-based deep clustering approaches leverage the trained encoder layers to transform the original data input into a lower-dimensional embedded representation. Recently, a deep embedding clustering (DEC) [12] method was introduced and then extended through a number of contributions [33,35,36,37,38,39]. One should mention that the DNN architecture in DEC [12] consists of a deep stacked autoencoder (SAE) network intended to automatically learn the feature representations through nonlinear data embedding. Note that the autoencoder is fine-tuned in order to minimize the reconstruction loss. The authors reported DEC accuracies of 84.3% and 75.63% using MNIST [40] and REUTERS [41] datasets, respectively. Later, DEC was adopted as a baseline for the empirical comparison of several deep clustering approaches [34,42,43]. Furthermore, other deep clustering works were proposed to address DEC limitations [12]. Namely, Improved Deep Embedded Clustering (IDEC) [38] was outlined as a DEC variant that maintains the valuable local structure of the data by freezing the decoder layers. Moreover, IDEC integrates and jointly optimizes the autoencoder’s reconstruction and clustering losses. In fact, keeping the decoder layers by IDEC helps in avoiding feature space distortion by clustering loss. Pursuing IDEC, several works take advantage of the decoder part of the autoencoder [10,44,45].

Specifically, DCEC was introduced in [32] as an extension of IDEC [38] for image clustering applications. DCEC relies on a convolutional autoencoder (CAE) as a deep network. This extension targets feature learning and embedding in an end-to-end-fashion. This contribution makes the model more suitable for clustering image data. Furthermore. Following DCEC, the Deep Continuous Clustering (DCC) approach was outlined in [34] to address two key limitations. Specifically, it relaxed the needs to (*i*) predefine the number of clusters for classical center-based approaches, and (*ii*) use discrete objective reconfigurations during optimization. DCC tackled these issues by optimizing a continuous objective using scalable gradient-based solvers. Later, another CAE-based deep clustering algorithm was proposed in [46]. It relaxed the need for pre-setting the number of clusters by adopting density-based distributions to discover homogenous clusters.

The authors in [24] observed that DEC [12] does not exploit prior knowledge to guide the learning process. In particular, they extended DEC [12] and proposed a new scheme of Semi-Supervised Deep Embedded Clustering (SDEC) to overcome this limitation. Specifically, SDEC incorporates pairwise constraints in the feature learning process. Its overall loss function is composed of two parts: the first consists of an unsupervised KL divergence loss, while the second part represents a semi-supervised loss that denotes the consistency between the learned representation with the prior information that is formed as pairwise constraints. SDEC’s best performance attained 86.11% in clustering accuracy and 82.89% in NMI for the MNIST image dataset. Since then, Semi-Supervised Deep Embedded Clustering (SS-DEC) has become a growing research field [47,48,49].

An earlier semi-supervised approach that employs supervision as constraints to learn the clustering metric was introduced in [50]. One should note that the proposed DC-SSDEC differs in two key aspects: (i) It uses deep neural networks to learn relevant feature representations and uncover hidden data partitions. In contrary, the approach in [50] that is not deep neural network-based uses hand-crafted features to encode the data into numerical vectors. (ii) The side information is incorporated into a DC-SSDEC objective function as a reward and penalty terms to govern the coherence between the learned representation and side information. As for the study in [50], the constraints are used to learn a Mahalanobis distance measure, and then cluster the data according to that metric (i.e., under similarity and dissimilarity constraints).

Recently, a semi-supervised deep embedding approach named SC-DEC was presented in [17]. SC-DEC leverages a deep neural network architecture and generates fuzzy membership degrees that better reveal the data partitioning. In particular, SC-DEC uses side information and formulates it as a set of soft pairwise constraints to steer the machine learning process. This supervision is expressed using relaxed “should-link” pairwise constraints. Such constraints determine whether the pairs of data instances should be assigned to the same or different cluster(s). In fact, the clustering task in SC-DEC was formulated as an optimization problem via the minimization of a designed objective function. SC-DEC reported clustering accuracy of 92.11% on MNIST, 76.62% on USPS, and 91.65% on STL-10 datasets, outperforming other relevant models. However, the effect of dual pairwise constraints was not investigated in SC-DEC. Moreover, studying the model was limited to benchmark datasets. In fact, the proposed DC-SSDEC differs from SC-DEC [17] in the prior knowledge considered to guide the clustering process. Specifically, soft “shouldNot-link” constraints are further designed to encode side information intended to further attend the clustering process. Additionally, unlike [17], which used only benchmark datasets, DC-SSDEC was tested on real-world datasets to solve factual problems. In particular, the Chest X-Ray dataset images were used for pneumonia detection. Moreover, DermaMNIST and American Digits Sign Language datasets were used to further investigate the proposed contributions. Table 1 below shows a summary of semi-supervised deep clustering works.

## 3. Dual-Constraint-Based Semi-Supervised Deep Clustering Approach

**Figure 1:**
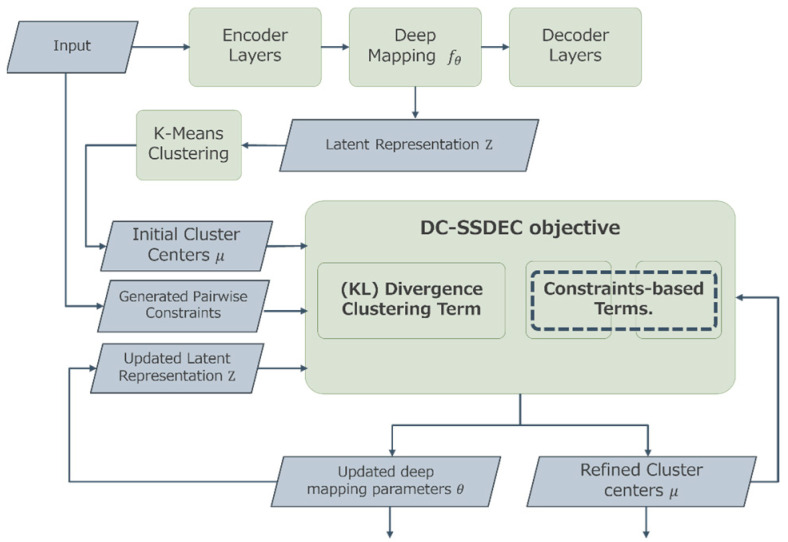
Overview of the proposed DC-SSDEC approach.

The proposed Dual-Constraint-based Semi-Supervised Deep Clustering (DC-SSDEC) approach utilizes a deep neural network architecture to carry out feature learning and clustering using fuzzy membership values. Specifically, the intended clustering problem is expressed as the optimization of a novel objective function. This function is designed to simultaneously uncover the hidden data clustering and optimize the deep neural network model. Additionally, the supervision information is integrated into the objective function as soft pairwise constraints, aiming to guide the partitioning process without being strictly enforced. Moreover, the data partition is represented using fuzzy membership functions to reflect the natural data grouping into homogeneous clusters. The proposed DC-SSDEC considers “should-link” and “shouldNot-link” pairwise constraints. As shown in Figure 1, DC-SSDEC relies on two main components: (*i*) an autoencoder AE architecture that learns the distinctive representation of the original data, and (*ii*) an objective function that performs the clustering tasks as advised.

As shown in Figure 1, the data input first passes through the first component, where the autoencoder layers are trained to initialize its parameters and learn the embedded data representation. The learned latent representation is then processed by a K-means clustering layer for cluster centers initialization. Subsequently, along with the generated pairwise constraints, the latent representation undergoes the second component (DC-SSDEC objective), where the deep network parameters and cluster centers are jointly optimized. This process continues until a specified threshold is met.

Let $X={\left\{x_{i}\right\}}_{i=1}^{n}$ be the unlabeled dataset that represents the input to the devised clustering algorithm. Accordingly, $x_{i}\;\in$ ${\mathbb{R}}_{d}$ constitute the data instances, and d represents the dimensions size of the original feature space. Rather than conducting the clustering task directly in the original data space, we define a nonlinear mapping function $f_{\theta }$ to transform the original input data X into a latent feature space Z; $f_{\theta }:X\;\to Z$, where θ represents the corresponding learnable parameters. One should note that the dimensionality of the embedded space Z is considerably lower than the dimensionality of the original data space. Accordingly, the primary purpose of the proposed DC-SSDEC algorithm is to learn an accurate data clustering in the latent feature space Z. This goal is mainly achieved using unlabeled data as well as some infused supervision. Specifically, it aims at partitioning the data into k clusters, where each cluster is defined using a $d^{\prime }$-dimensional centroid $\mu_{j=1,\ldots ,\;k}$. Note that $d^{\prime }$ represents the dimensionality of the embedded space Z. Basically, we intend to find a cluster-friendly $f_{\theta }$ such that the learned parameters are biased towards the clustering task and the available prior knowledge. Further details on the autoencoder architecture can be found in [17].

**Figure 2:**
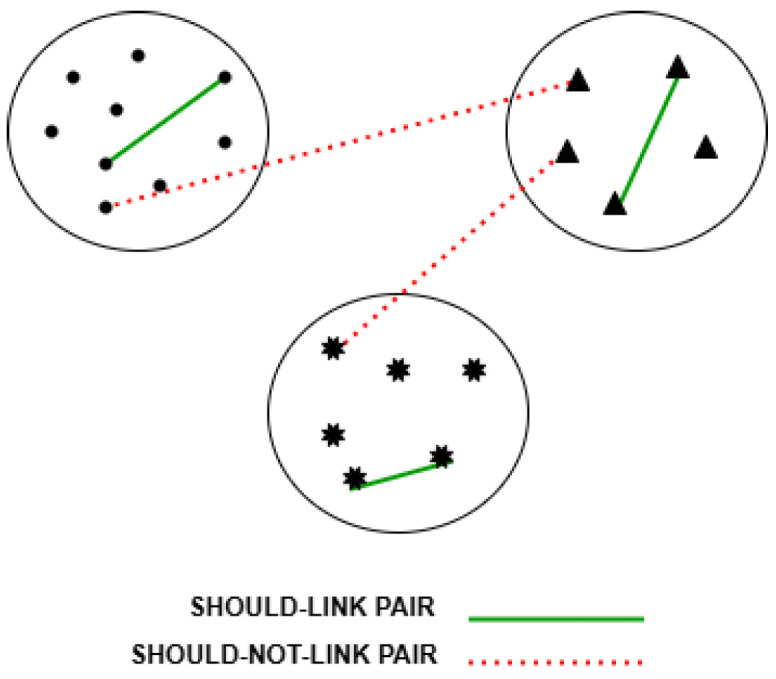
Illustration of the “should-link” and “shouldNot-link” pairwise constraints. Different symbols (circle, triangle and star) denote different clusters.

To guide the learning process, and escape local minima, side information is considered by the proposed DC-SSDEC. Specifically, it is expressed as two sets of pairwise constraints: (*i*) a set of “should-link” pairwise soft constraints denoted by S= {$\left(x_{i},\;x_{k}\right)$: $x_{i}$ and $x_{k}$ should be assigned to the same cluster, 1≤i, j≤N}; and (*ii*) a set of “shouldNot-link” soft pairwise constraints denoted by Sℕ= {$\left(x_{i},\;x_{k}\right)$: $x_{i}$ and $x_{k}$ should not be assigned to the same cluster, 1≤i, j≤N}. Figure 2 shows an illustrative example of the considered pairwise constraints links.

Typically, the supervision information is associated with a subset of the dataset only. One should note that these soft constraint sets are preset and produced from the dataset in a random way. In particular, the pairwise constraints are generated with a size equal to β×N, where β is the (ratio) of the pairwise constraints to the dataset size N. Specifically, for every pair of data points randomly drawn, we check their corresponding ground-truth labels. Accordingly, if the ground-truth labels of the samples pair are similar, a “should-link” pairwise link is generated between these samples and represented by 1. Otherwise, the pairwise link is represented by 0. Conversely, an opposing scenario takes place for the “shouldNot-link” pairwise link generation.

In this research, the clustering task is formulated as an optimization problem. Specifically, the proposed DC-SSDEC objective function encloses “should-link” and “shouldNot-link” constraints terms within the clustering objective function. More specifically, we designed the objective function below to learn the data partition along with its optimal parameters:

(1) $$J=\overset{n}{\underset{i=1}{\sum }}\overset{C}{\underset{j=1}{\sum }}p_{ij}log\frac{p_{ij}}{q_{ij}}-\gamma_{1}\left[\underset{x_{r},x_{k}\;\in \;S}{\sum }\overset{C}{\underset{j=1}{\sum }}q_{x_{r\;}j\;}^{m}q_{x_{k\;}j\;}^{m}\right]+\gamma_{2}\left[\underset{x_{r},x_{k}\;\in \;S\mathbb{N}}{\sum }\overset{C}{\underset{j=1}{\sum }}q_{x_{r\;}j\;}^{m}q_{x_{k\;}j\;}^{m}\right]$$

where n is the number of data points and C represents the number of clusters. On the other hand, m denotes the fuzziness parameter (a constant > 1) that reflects the fuzzy-based representation of the data clusters. Finally, S and Sℕ are the sets that represent the considered soft constraints. One should mention that the constraint term weights $\gamma_{1}$, $\gamma_{2}$ in (1) are the preset trade-off factors that balance the influence of the second and third terms (i.e., the constraint-based terms). Specifically, they balance the amount of penalty applied on the data batch in response to clustering errors. Additionally, $q_{ij}$ in (1) represents the soft assignment of the embedded data instances to the clusters.

The first term in (1) is inherited from the unsupervised deep embedded clustering term of DEC [12], which is expressed as the Kullback–Leibler (KL) divergence [67] based on the computed soft assignments Q to the computed auxiliary target distribution P. On the other hand, the second and third terms are the constraint-based terms that are intended to learn the compact, fuzzy-based clusters with the supervision guidance. This supervision is in the form of “should-link” and “shouldNot-link” soft pairwise constraints, respectively. In other words, the second and third terms are intended to reward/penalize the model for correct clustering, i.e., assigning a high membership value to a “should-link” pair of samples, to the same cluster.

Furthermore, the soft assignment value $q_{ij}$ in (1) is calculated using Student’s t-distribution as the kernel to compute the similarity between embedded point $z_{i}$ and cluster center $\mu_{j}$:

(2) $$q_{ij}=\frac{{\left(1+\|z_{i}-\mu_{j}{\|}^{2}/\alpha \right)}^{-\frac{\alpha +1}{2}}}{\sum_{j^{\prime }}{\left(1+\|z_{i}-\mu_{j}{\|}^{2}∕\alpha \right)}^{-\frac{\alpha +1}{2}}}\;,$$

where $z_{i}$ = $f_{\theta }$($x_{i\;}$) represents the embedded representation of $x_{i}$. Additionally, $\mu_{j}$ is the *j*th cluster center in the latent data space, ‖ . ‖ denotes the L2-norm and $\mu_{j^{\prime }}$ refers to all cluster centroids including the one in the numerator. The denominator is for the purpose of normalization, so that $q_{ij}$ values per data point can sum to one. Furthermore, α is the degrees of freedom of the Student’s t-distribution. We set α = 1 for all experiments. Hence, $q_{ij}$ is computed as follows:

(3) $$q_{ij}=\frac{{\left(1+\|z_{i}-\mu_{j}{\|}^{2}\right)}^{-1}}{\sum_{j^{\prime }}{\left(1+\|z_{i}-\mu_{j}{\|}^{2}\right)}^{-1}}$$

Additionally, $p_{ij}$ in (1) represents the auxiliary target distribution used to update the deep mapping $f_{\theta }$ and refine the cluster centroids. This update is implemented through learning from the current high confidence assignments in a self-training manner. Moreover, we compute $p_{ij}$ based on $q_{ij}$ from (3) by first squaring $q_{ij\;}$ and subsequently normalizing it based on cluster-wise frequency, as detailed below:

(4) pij=qij2fj∑j′qij′2fj′

where $f_{j}=\sum_{i}q_{ij}$ are the soft cluster frequencies and $f_{j^{\prime }}$ are the soft frequencies for all clusters, including the one in the numerator. Unlike hard clustering, fuzzy-based clustering approaches allow every data instance to belong to every cluster. This adds more flexibility to the proposed solution and facilitates the partitioning of data with vague boundaries. Typically, the membership value should be between 0 to 1, where 0 means the data instance does not belong to a specific cluster and 1 means it entirely belongs to that cluster. Moreover, the membership values of a data instance should sum up to 1. Fuzzy clustering is also known as probabilistic clustering. In fact, the fuzziness is introduced in DC-SSDEC by incorporating the fuzziness parameter m (1 ≤m) in the objective function to regulate the degree of data sharing between fuzzy clusters. On top of the AE encoder layers of the DC-SSDEC model, a custom clustering layer is added. Typically, it converts the data sample (the embedded features) to a soft label, i.e., a vector that reflects the fuzzy probability degree of the sample belonging to each cluster. This probability is computed with Student’s t-distribution.

Furthermore, in contrast to the rigid “must-link” and “cannot-link” constraints, the proposed formulation incorporates soft constraints. These constraints function as a reward for correctly clustering a data point, making this approach more suitable for the imprecise labeling commonly found in real-world datasets. The soft constraint term within the objective function governs the alignment between the learned representations and the clustering guided by the provided supervision.

Concretely, for a certain cluster j, $x_{i}$, $x_{k}\in \;S$ and given that the membership q has a fuzzy probabilistic value (q∈ [0, 1]): if $q_{ij}$ and $q_{kj}$ are both high, then the value of the resulting constraints term becomes high, thus promoting the minimization of the objective function; if $q_{ij}$ and $q_{kj}$ are both low, then the value of the resulting constraints term becomes quite low, thus having a neutral effect on the total of the objective function; if $q_{ij}$ is low and $q_{kj}$ is high (or vice versa), then the value of the resulting constraints term becomes relatively low, thus obstructing the objective function minimization.

The minimization of the objective function in (1) is preceded by the pretraining of an autoencoder AE for DNN parameter initialization. The AE training is performed based on the minimization of the following objective function:

(5) $$J_{AE}=\overset{n}{\underset{i=1}{\sum }}\|x_{i}-{\hat{x}}_{i}{\|}_{2}$$

where $\|x_{i}-{\hat{x}}_{i}{\|}_{2}$ is the Euclidean norm of the difference between data sample vector $x_{i\;}$ and its reconstructed sample vector ${\hat{x}}_{i}$. On the other hand, n is the size of the dataset.

The proposed algorithm minimizes the objective function in (1) iteratively and converges into the optimal clustering results, constrained by a tolerance threshold (%). Concretely, the proposed clustering approach encloses two phases: (*i*) A model parameter initialization where the DNN embedding parameters $f_{\theta }$ are initialized through the training of the deep autoencoder. Then, a K-means clustering algorithm [68] is deployed within the latent space Z for the initialization of the cluster centers $\mu_{j}$. (*ii*) A model parameter optimization intended to update the deep mapping $f_{\theta }$ and refine the cluster centers $\mu_{j\;}$. In this phase, the devised objective function is optimized through minimization. This minimization aims to learn from the upright high-confidence predictions. In particular, the objective iterates between the computation of the auxiliary target distribution and the minimization of the KL divergence objective term. Thus, the cluster assignment distribution is achieved through the minimization of the KL divergence with an auxiliary distribution.

## 4. Experiments

**Figure 3:**
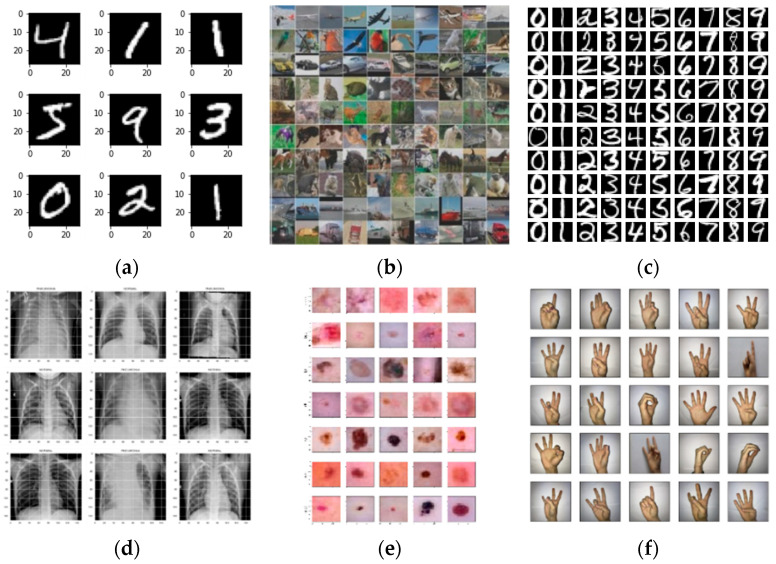
Sample images from: (**a**) MNIST, (**b**) STL-10, (**c**) USPS, (**d**) Pneumonia chest X-Ray, (**e**) DermaMNIST and (**f**) Digits-SL datasets.

The proposed DC-SSDEC was first assessed using three benchmark datasets widely adopted by deep clustering researchers. The first is the MNIST dataset [40], which comprises 70,000 grayscale images of handwritten digits. Each image is represented as a 28 × 28-pixel grid, resulting in a 784-dimensional feature vector. The second dataset is USPS [69], which consists of 9298 grayscale images of handwritten digits, each with a resolution of 16 × 16 pixels. Lastly, the STL-10 dataset [70], which contains 13,000 color images of natural scenes with a resolution of 96 × 96 pixels, was also considered in this research. Furthermore, to verify the performance of the proposed method in real-world applications, DC-SSDEC was associated with three real datasets. Namely, Chest X-Ray [71], DermaMNIST [72,73], and American Digits Sign Language [74] datasets were used in this research. Specifically, the Chest X-Ray radiographic imagery dataset [71] was considered for pneumonia disease detection and classification. More specifically, 5863 X-ray images from two categories—”Pneumonia” and “Normal”—were utilized. DermaMNIST [72,73] is a colored image collection including common pigmented skin lesions. The images size is 28 × 28 × 3 pixels. Finally, the American Sign Language digits dataset (Digits_SL) [74], which is relatively smaller, contains ten categories, representing the digits from 0 to 9. Table 2 reports the details of those datasets, along with the ground-truth information relevant to the intended clustering task. Sample images from the datasets used in this research are shown in Figure 3.

The performance of the proposed DC-SSDEC and those achieved by the challenger approaches were evaluated using standard metrics. The first one is the clustering accuracy (ACC), that measures the percentage of correctly grouped data points. It is computed using:

(6) $$ACC=\underset{m}{\max}\frac{\sum_{i=1}^{n}1\left\{l_{i}=m\left(c_{i}\right)\right\}}{n}$$

where $l_{i}$ is the ground truth, $c_{i}$ is the cluster assignment, n is the number of data points, and m spans over all possible one-to-one correspondences between the clusters and the predefined labels.

The second metric consists of the Normalized Mutual Information (NMI) measured using:

(7) $$NMI=\frac{MI\left(c,l\right)}{max\left(H\left(c\right),H\left(l\right)\right)}$$

where $H\left(l\right)$ represents the entropy of the ground truth l, $H\left(c\right)$ is the entropy of the cluster assignment c, and $MI\left(c,l\right)$ is the mutual information of c and l. NMI values range from 0 to 1, where a score of 1 implies that the two clustering outcomes are identical.

Regarding the non-linear mapping $f_{\theta }$, a fully connected stacked deep autoencoder network was adopted. For all experiments, the dimension size of the autoencoder layers is d–500–500–2000–10 dimensions, where d represents the dimensions of the input data space. Furthermore, the ReLU nonlinearity function [75] was selected as activation function for the internal layers of the autoencoder, with the exception of the input, output, and embedding layers. The initialization of the autoencoder weights was carried out through a greedy layer-wise pretraining. During the pretraining of the model using all datasets, “Adam” [76] was employed as the optimizer, with a default learning rate of 0.001, and the stopping threshold was set to 0.001. To initialize the cluster centers $\mu_{j}$, K-means clustering was run 20 times and the best result was exploited to set the initial centers.

### 4.1. Results Obtained Using Benchmark Datasets

These experiments aimed at assessing the performance of DC-SSDEC using benchmark datasets. Specifically, the objective function introduced in (1) was validated using MNIST [40], USPS [69], and STL-10 datasets. One should note that the model hyper-parameters went through a tuning process to obtain better settings and initialization. In particular, the optimal hyper-parameter values were set using a validation set and the accuracy as performance measure. Namely, we investigated the best number of pretraining epochs, the amount of supervision β, the fuzziness parameter m, the “should-link” constraint term weight $\gamma_{1}$, the “shouldNot-link” constraint term weight $\gamma_{2}$, and the data batch size. The following details the valid ranges for the model’s hyper-parameters: the size of the data batch size ϵ {x| x>0, x∈ℤ}, *β* ϵ {x| x>0, x ∈ℝ}, $\gamma_{1}$ and $\gamma_{2}$ ϵ {x| x∈ℝ} and m ϵ {x| x≥1, x∈ℝ}. In fact, these ranges were selected based on the best practices from the related literature and the considered benchmark datasets. Moreover, hyper-parameter tuning was carried out by allocating one experiment for each selected hyper-parameter value. After each hyper-parameter set of experiments, the value that resulted in the highest accuracy was saved and used for the rest of the experiments. Table 3 reports the hyper-parameter values that achieved the highest clustering accuracy for the proposed DC-SSDEC across the benchmark datasets.

The results achieved using DC-SSDEC were compared to those yielded by the relevant unsupervised and semi-supervised deep clustering algorithms. Specifically, the proposed method was compared with DEC [12], Semi-Supervised Deep Embedded Clustering (SDEC) [24], Improved Deep Embedded Clustering with Local Structure Preservation (IDEC) [38], K-means [77] applied to the deep embeddings of the considered datasets (DL + K-means), and the single-constraint based deep clustering approach (SC-DEC) [17]. One should note that the approach in [50] was not considered among the set of benchmark approaches because it failed to converge when associated with the datasets used in our experiments.

Table 4 reports the results achieved by DC-SSDEC, along with those obtained using the latter state-of-the-art methods along with MNIST, USPS, and STL-10 benchmark datasets. As can be seen in Table 4, the outperforming results obtained using DC-SSDEC showcase the advantage of enriching the prior knowledge by including the soft “shouldNot-link” constraints to guide the clustering process.

It can be noticed from Table 4 that DC-SSDEC overtook the other deep clustering approaches using all datasets in terms of the ACC metric. Regarding NMI, DC-SSDEC outperformed the relevant approaches, except SDEC [24], using the USPS [69] dataset. In fact, the results showed the positive effect of utilizing side information, as the proposed DC-SSDEC outperforms the unsupervised approach DEC [12]. Moreover, the fact that DC-SSDEC surpassed SC-DEC [17] demonstrates that coupling the set of “should-link” pairwise constraints with the set of “shouldNot-link” constraints let the clustering process escape local minima and yielded a better overall clustering accuracy. Although the designed objective function is different from the one in SC-DEC [17], the proposed dual constraints are expected to reduce the hypothesis and the search space, which reduces the risk of converging to local minima.

**Figure 4:**
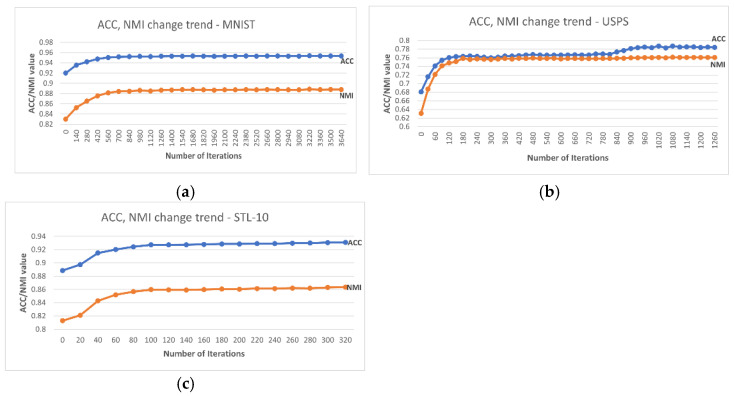
Accuracy and NMI trends recorded using DC-SSDEC and (**a**) MNIST, (**b**) USPS, and (**c**) STL-10 datasets.

Furthermore, at the individual class level, the lowest accuracy reported by DC-SSDEC increased by 11.9% in MNIST, 13.0% in USPS, and 10.5% in STL-10 compared to the single-constraint version in [17]. Additionally, DC-SSDEC exceeds the results obtained using DL + K-means for all three considered datasets. This demonstrates the importance of the joint optimization of deep embedding and clustering. The results also proved the importance of the fuzzy membership-based representations in enhancing the data partitioning. In fact, the proposed DC-SSDEC outperformed the non-fuzzy method SDEC [33]. In addition, Figure 4 shows the ACC and NMI trends for DC-SSDEC on the considered benchmark datasets. It is notable that the improvement in both ACC and NMI attainments is steadied after about 30% of the training time.

Further investigations show that the change in accuracy as a response to the increase in the β value was trivial for USPS and STL-10 datasets, as the difference between the highest and lowest accuracy did not exceed 0.14% and 0.81% for STL-10 and USPS datasets. On the other hand, this variance reached 8.84% for the MNIST dataset. The same observation holds for the effect of the weights γ2 and γ2 associated with both constraint terms. Additionally, the highest DC-SSDEC accuracy was achieved using the fuzzifier m set to 2 for the three datasets. One should also note that training DC-SSDEC on the USPS dataset using a 128-batch size resulted in better performance in comparison to using the default 256-batch size.

### 4.2. Results Obtained Using Real-World Datasets

In the next experiments, we assess the performance of DC-SSDEC using real-world datasets. Specifically, DC-SSDEC was deployed for medical image analysis and sign language recognition. In particular, these experiments included the assessment of DC-SSDEC performance when associated with two medical imagery datasets. Namely, the Chest X-Ray image dataset [71] was considered for pneumonia disease detection and the DermaMNIST image dataset [72,73] was used for dermatology disease detection. Moreover, similar experiments were carried out using the American Sign Language digits dataset, Digits_SL [74]. Furthermore, the results of DC-SSDEC using these datasets were evaluated against the results obtained from state-of-the-art approaches. Table 5 details the hyper-parameter values that yielded the best clustering accuracy for DC-SSDEC when associated with the Chest X-Ray dataset for pneumonia detection.

Generally, hyper-parameter tuning results show that the change in accuracy in response to the increased *β* value was not trivial, as the difference between the highest and lowest accuracy equaled 15.91%. Moreover, the effect of the size of constraints *β* on the performance of the proposed DC-SSDEC was relatively random. This may be influenced by the pairwise constraints generated, as their formation is randomized for each new value of β. Furthermore, the highest accuracy value for DC-SSDEC was gained when the value of the fuzzy parameter m equaled 2. This result is consistent with earlier experiments. In addition, training DC-SSDEC on the Chest X-Ray dataset using a 64-batch size yielded the highest results. This can be related to the small size of the dataset.

Table 6 reports the pneumonia detection performance achieved using DC-SSDEC and the considered state-of-the-art methods along with the Chest X-Ray dataset. As can be seen, improvements of approximately 10% and 7% were achieved by DC-SSDEC compared to the other methods, for the accuracy and NMI, respectively.

**Figure 5:**
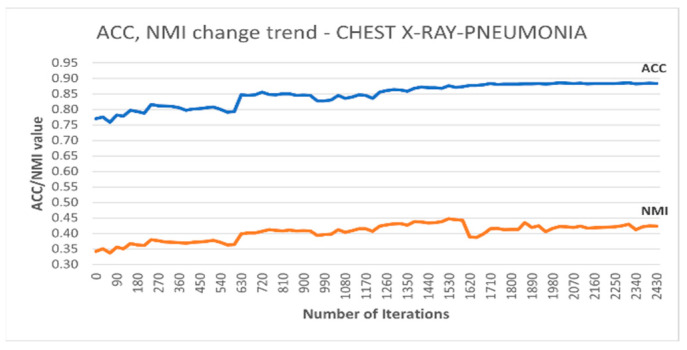
Trends recorded for the performance metrics achieved using DC-SSDEC on the Chest X-Ray dataset.

Additionally, Figure 5 depicts the accuracy and NMI trends for DC-SSDEC on the Chest X-Ray dataset. It is notable that the improvement in terms of both accuracy and NMI measures is steadied after around 60% of the training time.

In the following, we outline the results obtained using the proposed DC-SSDEC when associated with the DermaMNIST dataset [72,73] for dermatological disease detection. The assessment began with running a set of experiments to calibrate the relevant hyper-parameters. After tuning the model hyper-parameters to their best values in terms of clustering accuracy, we ran 20 experiments using these values. Hyper-parameter values that yielded the highest accuracy of DC-SSDEC for the DermaMNIST dataset are reported in Table 7. In particular, hyper-parameter tuning results show that with only a ratio of 0.3 of the pairwise constraints (*β* hyper-parameter), DC-SSDEC yielded the highest accuracy. This result is similar to the one obtained when evaluating DC-SSDEC on MNIST. Also, consistent with past experiments, the results show that tuning the fuzzifier to the value of 2 returns the highest accuracy on DermaMNIST.

The results achieved by DC-SSDEC in these experiments are reported in Table 8. The results show that our approach outperformed the other state-of-the-art approaches, with an increase of approximately 13.3% in terms of clustering accuracy. Furthermore, all of the considered methods yielded rather weak results in terms of the NMI score.

**Figure 6:**
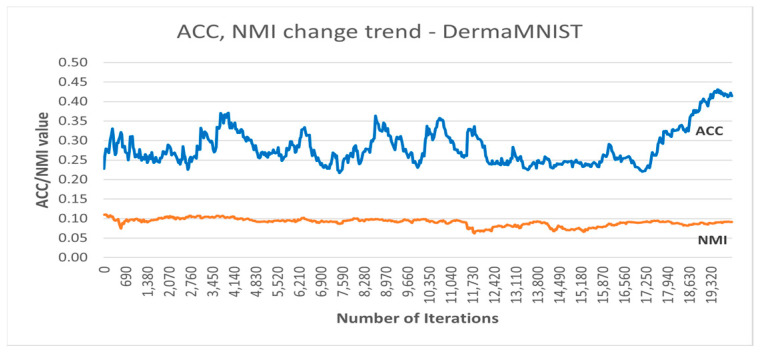
Accuracy and NMI trends recorded using DC-SSDEC and the DermaMNIST dataset.

Figure 6 shows the accuracy and NMI trends over the training iterations of the best DC-SSDEC run on the DermaMNIST dataset. As can be seen, after some fluctuation during the training, the accuracy improved by almost 19% from the initial accuracy recorded.

Hyper-parameter values that yielded the highest accuracy of DC-SSDEC on the Digits-SL dataset are reported in Table 9. Generally, the results of this hyper-parameter tuning show that the highest accuracy was attained at values of −1 and 1 for *γ*1 and *γ*2, respectively. This is consistent with the initial structure of the proposed objective function. Furthermore, the accuracy results had an inverse relationship with the data batch size adopted for pretraining, where training DC-SSDEC on the Digits-SL dataset using a 32-batch size yielded the highest results. This can be related to the small scale of the dataset.

Finally, Table 10 reports the results achieved by DC-SSDEC and the considered state-of-the-art approaches using the Digits_SL dataset [74] for sign language detection. The results shown in the table demonstrate the dominance of the proposed approach. Specifically, an improvement of approximately 9.3% over other methods was recorded in terms of clustering accuracy.

Also, an improvement near 8.77% was achieved by DC-SSDEC in terms of the NMI metric. In addition, the relatively low accuracy values in Table 10 can be attributed to the small size of the highly dimensional Digits_SL dataset.

**Figure 7:**
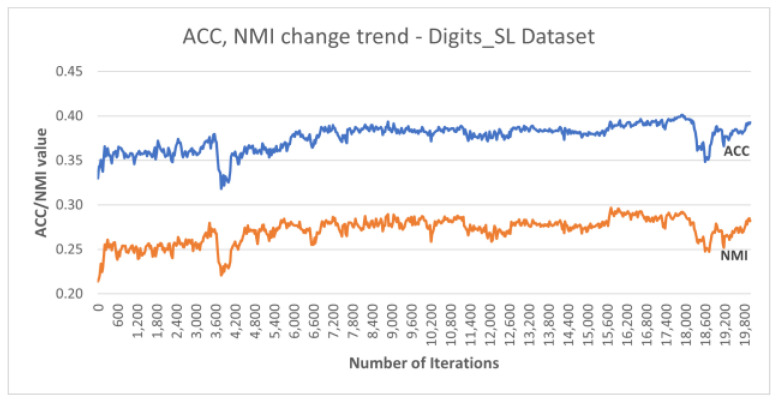
Performance trends recorded for DC-SSDEC using the Digits_SL dataset.

Additionally, Figure 7 plots the accuracy and NMI trends over the training iterations for the best run of DC-SSDEC using the Digits_SL dataset. It is notable that both metrics improved by 6% from the initial accuracy recorded, with some fluctuation throughout the training process.

## 5. Conclusions

This research introduced a novel semi-supervised deep clustering approach that associates deep neural networks and fuzzy membership functions for an improved clustering performance. The proposed Dual-Constraint-based Semi-Supervised Deep Clustering approach, DC-SSDEC, exploits “should-link” and “shouldNot-link” pairwise soft constraints to guide the clustering process. In particular, the intended clustering task was expressed as an optimization problem through the minimization of a newly objective function. Comprehensive experiments were conducted to validate and assess DC-SSDEC performance. The results obtained demonstrated DC-SSDEC outperformance compared to relevant deep clustering approaches. Specifically, when evaluated against the state-of-the-art approaches, DC-SSDEC yielded competitive clustering results using the STL-10 dataset and exceeded the performance of the state-of-the-art methods using MNIST, USPS, Chest X-Ray, DermaMNIST, and Digits-SL datasets. Typically, the obtained results showcased the added value of the proposed formulation of the soft pairwise constraints considered. Specifically, enriching the set of “should-link” pairwise constraints with another set of “shouldNot-link” constraints further enhanced the clustering performance. Additionally, the superior performance of DC-SSDEC in comparison to (DL + K-means) on all datasets demonstrated the efficiency of the proposed simultaneous optimization of (*i*) the clustering objective function and (*ii*) the deep network loss function.

In fact, whereas crisp clustering mandates that each data sample be assigned to a single cluster, soft clustering allows data points to belong to multiple clusters simultaneously. This flexibility enables soft clustering to handle clusters with unclear boundaries more effectively. In contrast, crisp clustering methods often struggle to identify clusters with irregular shapes, making them more prone to local minima. As a result, the performance of DC-SSDEC highlighted the positive impact of adopting soft clustering, in comparison to crisp clustering methods. As an application of soft clustering, fuzzy membership representations boosted the performance of the proposed approach over the non-fuzzy approach, SDEC [24], for all datasets.

The proposed approach, DC-SSDEC, can be coupled with any image datasets. In particular, medical imaging analysis, information security purposes such as surveillance and intrusion detection, and Content-Based Image Retrieval (CBIR) represent typical applications for the proposed approach. Furthermore, we should state some limitations of the proposed approach, such as its sensitivity to the predetermined number of clusters and its dependence on trial-and-error methods for hyper-parameter tuning. To address these issues and improve performance, future research directions are suggested: (*i*) automating cluster number determination by incorporating an additional term into the proposed objective function; (*ii*) exploring advanced heuristics for selecting hyper-parameters (*β*, *γ*); (*iii*) expanding the range of tunable hyper-parameters, such as those for autoencoder settings and the optimizer’s learning rate; (*iv*) managing noise and outliers using a possibilistic logic-based clustering approach; (*v*) incorporating weights into the labeled instances associated with the pairwise constraints; and (*vi*) analyzing the computational complexity of the algorithm.

## Figures and Tables

**Table 1:** Summary of related semi-supervised deep clustering works.

No	Architecture	Citation	Date	Supervision Method	Clustering Method	Application
1	Dual Autoencoder	[13]	2024	Applying a semi-supervised autoencoder layering approach	Attributed graph Clustering	General Clustering
2	Stacked Restricted Boltzmann Machines (RBMs)	[51]	2015	Must-link and cannot-link pairwise constraints	Maximum Margin Clustering	General clustering
3	Siamese networks	[52]	2022	Must-link and cannot-link pairwise constraints	K-means	General clustering
4	Text Convolutional Siamese Network	[26]	2020	Must-link and cannot-link pairwise constraints	K-means	Text (document) clustering
5	Long Short-Term Memory (LSTM) architecture	[53]	2016	Instance-level constraints	K-means	Text (document) clustering
6	Convolutional Neural Networks (CNNs)	[53]	2016	Instance-level constraints	K-means	Text (document) clustering
7		[54]	2018	A small amount of labeled image annotations	Maximum Margin Clustering with hierarchical-based clustering	Classification-oriented clustering
8		[47]	2019	Instance-level constraints	K-means + KLD	Infant Brain MRI tissue segmentation
9		[55]	2020	Labeled triplets constraints	Graph-based clustering	General clustering
10		[14]	2024	Clustering constraint layer is designed to improve the clustering efficiency.	graph clustering	Image Clustering
11	Specific introduced DNN architecture	[56]	2020	Use Siamese network to compute pairwise constraints loss between two same weighted embedding systems	K-means	General clustering
12	Convolutional Autoencoder (CAE)	[15]	2023	A small amount of prior information	Density-based clustering	Clustering non spherical datasets with regular clusters
13		[16]	2022	Must-link and cannot-link pairwise constraints	K-means	Clustering multi-view data
14		[57]	2019	Labeled normal samples and labeled anomalies	N/A *	General anomaly detection
15		[27]	2020	Must-link and cannot-link pairwise constraints	K-means + KLD	General clustering
16		[20]	2019	Prior on class proportions	Objective function of regularized optimal transport	Clustering datasets with well-balanced classes
17		[58]	2021	Cross-entropy based self-labeling of samples	K-means + KLD	Classification-oriented clustering
18	Wasserstein Autoencoder and Generative network	[59]	2020	Instance-level constraints	Generative-based clustering	Generative purposes
19	Stacked Autoencoder (SAE)	[60]	2023	Set of neighborhoods where active learning approach uses parallel clustering layers as a source for selecting useful prior data	N/A	General clustering
20		[16]	2022	Must-link and cannot-link pairwise constraints	K-means	Clustering multi-view data
21		[49]	2019	Labeled triplets constraints	K-means + KLD	General clustering
22		[25]	2020	Must-link and cannot-link pairwise constraints	K-means + KLD	Anomaly detection for semantic frame induction
23		[24]	2019	Must-link and cannot-link pairwise constraints	K-means + KLD	General clustering
24		[17]	2023	Should-link pairwise constraints	K-means + KLD	General clustering
25	Stacked, Denoising Autoencoder	[35]	2021	Standard and complex constraints, including continuous values and higher-level domain knowledge	K-means + KLD	Clustering scenarios with noisy constraints
26		[61]	2021	The soft membership affinity	K-means + KLD	General clustering
27		[62]	2019	Continuous values (triplet constraints), instance difficulty constraints, and balancing constraints at the cluster level	K-means	Clustering that deals with noisy constraints
28	Denoising Autoencoder	[18]	2023	Must-link and cannot-link pairwise constraints	KLD clustering with intention guider	Text (document) clustering
29		[63]	2021	The supervision is incorporated using existing label information, through a voting mechanism that adapts labels dynamically	K-means + KLD	General clustering
30		[48]	2020	Instance-level constraints	K-means + KLD	Electronic health record (EHR) patient cohorts—clustering
31	Deep Autoencoder	[64]	2021	Must-link and cannot-link pairwise constraints	K-means + KLD	Clustering large Scale datasets
32		[45]	2021	The target distribution is obtained using new fuzzy net, not using self-training strategy	K-means + KLD	General clustering
33		[65]	2021	Instance-level constraints	K-means and spectral clustering	Identifying Cross-Cancer Similar Patients
34		[21]	2024	The pairwise constraints; must-link and cannot-link pairs are utilized to determine the thresholds for similarity and dissimilarity between samples.	Distance-based clustering	General clustering
35	Siamese Autoencoder	[66]	2019	Must-link and cannot-link pairwise constraints. Additionally, the supervision is refined by analyzing the losses associated with each pair to refine the set of constraints	A distance measurement between the clustering representation of pairs of data points. Pairs are assembled from a connectivity graph, which we construct using a mutual KNN (MKNN) connectivity between data points in the initial space X	General clustering

* Not Available.

**Table 2:** Description of the datasets considered.

Dataset	No. Samples	No. Classes	No. Dimensions
MNIST	70,000	10	784
USPS	9298	10	256
STL-10	13,000	10	4096
Chest X-Ray Images	5856	2	8192
DermaMNIST	10,015	7	2352
Digits-SL	2062	10	4096

**Table 3:** Hyper-parameter values that yielded the highest accuracy of DC-SSDEC for the different datasets.

Hyper-Parameter	MNIST	USPS	STL-10
Number of AE pretrain epochs	400	200	10
Ratio of constraints *β*	0.3	1	1
Fuzzifier *m*	2	2	2
Constraint Term weight *γ*1	−10	−1	1
Constraint Term weight *γ*2	10	−1	100
Data batch size	256	128	256

**Table 4:** Clustering results in terms of ACC and NMI, achieved using the different approaches and datasets.

	**MNIST**		**STL-10**		**USPS**	
**Approach**	**ACC %**	**NMI %**	**ACC %**	**NMI %**	**ACC %**	**NMI %**
DL + K-means	86.83	76.89	86.94	78.84	70.93	68.44
DEC	84.30	NA	35.90	NA	NA	NA
SDEC	86.11	82.89	38.86	32.84	76.39	**77.68**
IDEC	83.84	77.88	NA	NA	72.69	71.13
SC-DEC	92.11	84.01	91.65	84.85	76.62	75.31
Proposed DC-SSDEC	95.36 *	**88.79**	**93.09**	**86.35**	**78.44**	76.06

* Bold highlights the highest accuracy.

**Table 5:** Hyper-parameter values that yielded the highest accuracy for DC-SSDEC and the Chest X-Ray dataset.

Hyper-Parameter	Value
Number of AE pretraining epochs	20
Ratio of constraints *β*	1
Fuzzifier *m*	2
Constraint Term weight *γ*1	1
Constraint Term weight *γ*2	1
Data batch size	64
Embedded feature dimensions size	10

**Table 6:** Clustering results obtained using the Chest X-Ray dataset.

Approach	ACC %	NMI %
Simple K-means	76.83	34.12
DL + K-means	77.29	34.77
DEC	71.16	28.81
SDEC	72.97	0.00
IDEC	75.99	33.78
DC-SSDEC	88.40 *	**42.398**

* Bold highlights the highest accuracy.

**Table 7:** Hyper-parameter values that yielded the highest accuracy of DC-SSDEC for the DermaMNIST dataset.

Hyper-Parameter	Value
Number of AE pretraining epochs	30
Ratio of constraints *β*	0.3
Fuzzifier *m*	2
Constraint Term weight *γ*1	1
Constraint Term weight *γ*2	1
Data batch size	64
Embedded feature dimensions size	10

**Table 8:** Clustering results obtained using the DermaMNIST dataset.

Method	ACC %	NMI %
Simple K-means	28.12	8.37
DL + K-means	22.49	9.66
DEC	27.61	7.70
IDEC	27.01	**10.82**
SDEC	26.83	8.01
DC-SSDEC	41.42 *	9.15

* Bold highlights the highest accuracy.

**Table 9:** Hyper-parameter values that yielded the highest accuracy of DC-SSDEC for the Digits-SL dataset.

Hyper-Parameter	Value
Number of AE pretraining epochs	80
Ratio of constraints *β*	2
Fuzzifier *m*	2
Constraint Term weight *γ*1	−1
Constraint Term weight *γ*2	1
Data batch size	32
Embedded feature dimensions size	10

**Table 10:** Clustering results obtained using the Digits_SL dataset.

Method	ACC %	NMI %
Simple K-means	27.64	15.34
DL + K-means	29.97	19.43
DEC	10.09	0.00
IDEC	16.44	4.06
SDEC	10.09	0.00
DC-SSDEC	39.28 *	**28.20**

* Bold highlights the highest accuracy.

## Data Availability

Publicly available datasets were analyzed in this study. The MNIST data can be found at https://www.kaggle.com/datasets/hojjatk/mnist-dataset/data. The USPS data can be found at https://www.csie.ntu.edu.tw/~cjlin/libsvmtools/datasets/multiclass.html#usps. The STL-10 data can be found at https://cs.stanford.edu/~acoates/stl10/. The Chest X-Ray data can be found at https://www.kaggle.com/datasets/paultimothymooney/chest-xray-pneumonia. The DermaMNIST data can be found at https://zenodo.org/records/10519652. The Digits_SL data can be found at https://www.kaggle.com/datasets/ardamavi/sign-language-digits-dataset. (All data sources above were accessed on 22 January 2025.)
